# Association of Severe Bronchiolitis during Infancy with Childhood Asthma Development: An Analysis of the ECHO Consortium

**DOI:** 10.3390/biomedicines11010023

**Published:** 2022-12-22

**Authors:** Makiko Nanishi, Aruna Chandran, Xiuhong Li, Joseph B. Stanford, Akram N. Alshawabkeh, Judy L. Aschner, Dana Dabelea, Anne L. Dunlop, Amy J. Elliott, James E. Gern, Tina Hartert, Julie Herbstman, Gurjit K. Khurana Hershey, Alison E. Hipwell, Margaret R. Karagas, Catherine J. Karr, Leslie D. Leve, Augusto A. Litonjua, Cindy T. McEvoy, Rachel L. Miller, Emily Oken, T. Michael O’Shea, Nigel Paneth, Scott T. Weiss, Robert O. Wright, Rosalind J. Wright, Kecia N. Carroll, Xueying Zhang, Qi Zhao, Edward Zoratti, Carlos A. Camargo, Kohei Hasegawa

**Affiliations:** 1 Department of Emergency Medicine, Massachusetts General Hospital, Harvard Medical School, Boston, MA 02114, USA; 2 Department of Epidemiology, Johns Hopkins Bloomberg School of Public Health, Johns Hopkins University, Baltimore, MD 21218, USA; 3 Department of Family and Preventive Medicine, University of Utah, Salt Lake City, UT 84112, USA; 4 Department of Civil and Environmental Engineering, Northeastern University, Boston, MA 02115, USA; 5 Departments of Pediatrics, Hackensack Meridian School of Medicine, Albert Einstein College of Medicine, Bronx, NY 10461, USA; 6 Lifecourse Epidemiology of Adiposity and Diabetes (LEAD) Center, University of Colorado Anschutz Medical Campus, Aurora, CO 80045, USA; 7 Department of Gynecology and Obstetrics, Emory University School of Medicine, Atlanta, GA 30307, USA; 8 Avera Research Institute & Department of Pediatrics, University of South Dakota School of Medicine, Sioux Falls, SD 57069, USA; 9 Department of Pediatrics, University of Wisconsin School of Medicine and Public Health, Madison, WI 53726, USA; 10 Departments of Medicine and Pediatrics, Vanderbilt University Medical Center, Nashville, TN 37232, USA; 11 Environmental Health Sciences, Mailman School of Public Health, Columbia University, New York, NY 10027, USA; 12 Division of Asthma Research, Cincinnati Children’s Hospital, Department of Pediatrics, University of Cincinnati, Cincinnati, OH 45221, USA; 13 Department of Psychiatry, University of Pittsburgh, Pittsburgh, PA 15260, USA; 14 Department of Epidemiology, Geisel School of Medicine, Dartmouth College, Hanover, NH 03756, USA; 15 Department of Epidemiology, University of Washington, Seattle, WA 98195, USA; 16 Department of Environmental and Occupational Health Sciences, University of Washington, Seattle, WA 98195, USA; 17 Department of Pediatrics, University of Washington, Seattle, WA 98195, USA; 18 Prevention Science Institute, University of Oregon, Eugene, OR 97403, USA; 19 Division of Pediatric Pulmonary Medicine, Department of Pediatrics, University of Rochester Medical Center, Rochester, NY 14642, USA; 20 Department of Pediatrics, Oregon Health & Science University, Portland, OR 97239, USA; 21 Division of Clinical Immunology, Department of Medicine, Icahn School of Medicine, New York, NY 10029, USA; 22 Division of Chronic Disease Research Across the Lifecourse, Department of Population Medicine, Harvard Medical School, Harvard Pilgrim Health Care Institute, Boston, MA 02215, USA; 23 Division of Neonatal-Perinatal Medicine, University of North Carolina at Chapel Hill, Chapel Hill, NC 27559, USA; 24 Departments of Epidemiology and Biostatistics and Pediatrics and Human Development, Michigan State University, College of Human Medicine, East Lansing, MI 49503, USA; 25 Channing Division of Network Medicine, Department of Medicine, Brigham and Women’s Hospital, Boston, MA 02115, USA; 26 Department of Environmental Medicine and Public Health, Icahn School of Medicine at Mount Sinai, New York, NY 10029, USA; 27 Department of Preventive Medicine, The University of Tennessee Health Science Center, Memphis, TN 38163, USA; 28 Department of Medicine, Henry Ford Health, Detroit, MI 48202, USA

**Keywords:** asthma, bronchiolitis, children, cohort, generalizability, heterogeneity

## Abstract

**Objective**: Many studies have shown that severe (hospitalized) bronchiolitis during infancy is a risk factor for developing childhood asthma. However, the population subgroups at the highest risk remain unclear. Using large nationwide pediatric cohort data, namely the NIH Environmental influences on Child Health Outcomes (ECHO) Program, we aimed to quantify the longitudinal relationship of bronchiolitis hospitalization during infancy with asthma in a generalizable dataset and to examine potential heterogeneity in terms of major demographics and clinical factors. **Methods:** We analyzed data from infants (age <12 months) enrolled in one of the 53 prospective cohort studies in the ECHO Program during 2001–2021. The exposure was bronchiolitis hospitalization during infancy. The outcome was a diagnosis of asthma by a physician by age 12 years. We examined their longitudinal association and determined the potential effect modifications of major demographic factors. **Results:** The analytic cohort consisted of 11,762 infants, 10% of whom had bronchiolitis hospitalization. Overall, 15% subsequently developed asthma. In the Cox proportional hazards model adjusting for 10 patient-level factors, compared with the no-bronchiolitis hospitalization group, the bronchiolitis hospitalization group had a significantly higher rate of asthma (14% vs. 24%, HR = 2.77, 95%CI = 2.24–3.43, *p* < 0.001). There was significant heterogeneity by race and ethnicity (P_interaction_ = 0.02). The magnitude of the association was greater in non-Hispanic White (HR = 3.77, 95%CI = 2.74–5.18, *p* < 0.001) and non-Hispanic Black (HR = 2.39, 95%CI = 1.60–3.56; *p* < 0.001) infants, compared with Hispanic infants (HR = 1.51, 95%CI = 0.77–2.95, *p* = 0.23). **Conclusions:** According to the nationwide cohort data, infants hospitalized with bronchiolitis are at a higher risk for asthma, with quantitative heterogeneity in different racial and ethnic groups.

## 1. Introduction

Childhood asthma is a major public health problem in the U.S. Asthma affects approximately 8% of children (6 million), with racial and ethnic disparities, e.g., a higher prevalence in African-Americans [1,2,3] and children of Puerto Rican ethnicity [4]. These racial and ethnic disparities are widely recognized, with active research being carried out on the possible mechanisms. The literature has also demonstrated many risk factors for incident asthma, including genetic factors [5,6,7,8], a personal and family history of atopy [2,9,10], environmental exposures (e.g., acute respiratory infection, socioeconomic status) [11,12,13,14,15,16,17], and lifestyle (e.g., breastfeeding, prenatal smoking) [18,19,20,21,22].

Of these risk factors, acute lower respiratory tract infection (e.g., bronchiolitis) during infancy is the risk factor with the largest attributable fraction of the population in asthma. [23] Indeed, epidemiological studies have suggested that 20–40% of infants hospitalized for bronchiolitis (“severe bronchiolitis”) will subsequently develop childhood asthma [11,12,17,24,25,26,27,28]. However, these reports have potentially limited generalizability due to their assessment of populations with restricted demographic and geographical characteristics. Furthermore, little is known about the heterogeneity of the bronchiolitis–asthma relationship across different demographic and clinical subgroups.

To address this knowledge gap, we analyzed the data of 11,762 children from 53 cohort studies across the nation to (1) investigate the longitudinal relationship of severe bronchiolitis during infancy with the subsequent development of childhood asthma, and (2) determine the potential effect modifications by major demographic and clinical factors. The identification of infant subgroups at higher risk could lead to the development of more tailored prevention strategies for childhood asthma.

## 2. Materials and Methods

### 2.1. Study Design, Setting, and Participants

This is an analysis of data from the National Institutes of Health (NIH) Environmental influences on Child Health Outcomes (ECHO) program. ECHO is a nationwide consortium of 67 pediatric cohorts that aims to leverage its demographic and geographic heterogeneity and large sample size to address important research questions. [29] We excluded 14 cohorts because of the lack of exposure and outcome data. Data submitted by 53 cohorts funded through ECHO as of 31 August 2021 were used in the current analysis. Data analyses were conducted by the central ECHO Data Analysis Center (DAC), located at the Johns Hopkins Bloomberg School of Public Health (Baltimore, MD, USA). All participating cohorts had institutional review board (IRB) approval either through ECHO’s central IRB or from their local institutions; the work of the DAC was approved by the Johns Hopkins Bloomberg School of Public Health IRB.

The inclusion criteria for the current analysis were being aged ≥5 years at the last follow-up visit seen with information on hospitalizations in infancy (age <1 year) and data on asthma diagnosis, symptoms, and medication use. We excluded children with a diagnosis of asthma before the age of 1 year. In total, 53 cohorts contributed data to this analysis (Table S1 and Figure S1). Of these, one prospective cohort specifically enrolled 921 infants hospitalized with bronchiolitis [11] and followed those children prospectively. Additionally, other cohorts specifically enrolled 6296 children with a parental history of asthma and/or atopy. The remainder of the cohorts were general population cohorts or had enrollment criteria unrelated to bronchiolitis or asthma.

### 2.2. Exposure

The exposure of interest was a parent/caregiver report of hospitalization for bronchiolitis or bronchitis during infancy (<12 months of age). Age at hospitalization, if not collected directly, was imputed on the basis of the age at the study visit. If a study visit occurred after 12 months of age but collected data specifically regarding the infancy period, the age at hospitalization was imputed to be 6.0 months (the halfway point of the infancy age range).

### 2.3. Outcome Measure

The outcome of interest was a parent/caregiver report of provider-diagnosed asthma up to the age of 12 years. If the exact age of diagnosis was unavailable, age was estimated using the midpoint of the age between the first indication of having asthma and the visit prior to that. For children diagnosed before 5 years of age, inclusion as an outcome required an indication of ongoing asthma symptoms or medication use (e.g., bronchodilators, inhaled corticosteroids) at age 5 years or later [30]. Accordingly, all asthma cases in this analysis diagnosed between ages of 1 and 4 years represent children with ongoing asthma at age 5 years or later to avoid the inclusion of early “transient wheezers” misdiagnosed as having asthma [31].

### 2.4. Statistical Analyses

Descriptive statistics were used to describe and compare the major demographic and clinical characteristics between infants with and without bronchiolitis hospitalization. Time to incident asthma diagnosis was compared between the two groups using a Kaplan–Meier curve. Follow-up ended at the first visit at which the child was determined to have an incident diagnosis of asthma, at loss to follow-up (the last visit with an assessment of asthma status), or at censoring at the age of 12.9 years, whichever occurred first. Multiple imputations were used to impute missing data by fully conditional specification with a discriminant function method [32]. All variables were included in the imputation model to impute the following variables for 25 imputations: child’s race and ethnicity (<1%; non-Hispanic White, non-Hispanic Black, non-Hispanic Asian, non-Hispanic other race, and Hispanic), gestation age (13%; <37 weeks or ≥37 weeks), breastfeeding (44%; ever breastfed and never breastfed), child atopy history (52%; any history of provider-diagnosed food allergy, allergic rhinitis, and/or eczema), parental history of asthma (19%; any reported history of asthma in the biological mother, father, or both vs. no parental history of asthma), prenatal smoking (18%; ever having used tobacco any time during pregnancy), maternal age at childbirth (9%; <25 y, 25–40 y, and >40 y), and maternal education (10%; <high school, high school graduate or equivalent, and some college or above). Cohort ID was also included in the imputation model as a classification variable.

To estimate the association of bronchiolitis hospitalization in infancy with the rate of developing childhood asthma, multivariable Cox proportional hazard models were fitted using a random cohort effect, adjusting for the 10 potential confounders (sex, race and ethnicity, calendar year of childbirth, gestational age, breastfeeding, child’s atopy, parental asthma, prenatal smoking, maternal age, and maternal education), accounting for potential patient clustering within cohorts. We confirmed the validity of the assumption of proportional hazards by examining the log–log survival curve. These covariates were selected on the basis of clinical plausibility and a priori knowledge [2,11,33]. Additionally, we repeated the models without multiple imputation (complete case analysis).

To examine the potential effect modification on the bronchiolitis–asthma association, we tested for the interactions by a likelihood ratio test and repeated the multivariable Cox proportional hazard models, stratifying by nine major demographic and clinical factors, i.e., child’s sex, race and ethnicity, gestational age, breastfeeding, child’s atopy, parental asthma, prenatal smoking, maternal age at childbirth, and maternal education.

In the sensitivity analysis, we computed E-values to determine the robustness of causal inference against potential unmeasured confounding [34]. The E-value represents the minimum magnitude of association that a set of unmeasured confounders would need to have in order to fully explain the association of interest, conditional on the covariates. For example, an E-value of 2.0 means that the hazard ratio (HR) for the association of unmeasured confounders with both the exposure and outcome would have to be ≥2.0 to explain away the observed exposure–outcome association. A *p*-value of <0.05 was considered to be statistically significant. All analyses were conducted in SAS 9.4 (SAS Institute Inc., Cary, NC, USA) and R version 3.6.2.

## 3. Results

### 3.1. Patients’ Characteristics

Of 57,982 infants in the ECHO program, 11,762 were eligible for the current analysis (Figure S2). Overall, the median age at bronchiolitis hospitalization was 9 months (IQR = 7–11 months); 53% were male; and 53% were non-Hispanic White, 18% were non-Hispanic Black, and 18% were Hispanic. Of the infants in the analytic cohort, 1130 (10%) were hospitalized for bronchiolitis, i.e., severe bronchiolitis (Table 1). The number of missing data is displayed in Table S2.

### 3.2. Associations of Severe Bronchiolitis with the Rate of Developing Childhood Asthma

**Figure 1:**
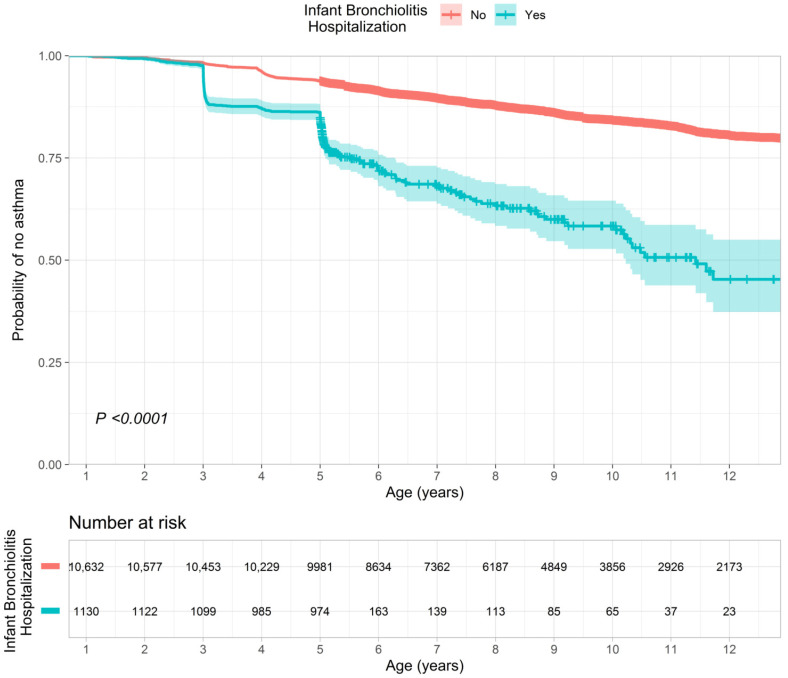
Kaplan–Meier curves for developing childhood asthma, according to bronchiolitis hospitalization (severe bronchiolitis).

**Figure 2:**
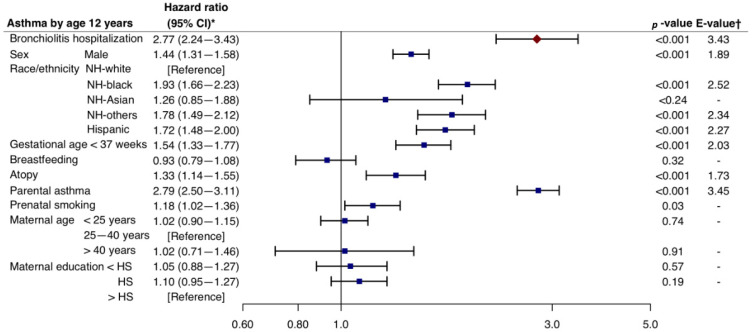
Associations of bronchiolitis hospitalization in infancy with the subsequent development of asthma. * The Cox proportional hazard model was adjusted for 10 potential confounders (sex, race and ethnicity, calendar year of childbirth, gestational age, breastfeeding, child’s atopy, parental asthma, prenatal smoking, maternal age at delivery, and maternal education), accounting for patient clustering by cohort. † The E-value (with its lower 95% CI bound) represents how strongly unmeasured confounder(s) would have to be associated with the exposure and outcome in order for the observed association to be independent. Abbreviations: CI, confidence interval; HS, high school; NH, Non-Hispanic.

Overall, 15% subsequently developed asthma by the age of 12 years (the mean follow-up time in this time-to-incident analysis was 7.5 years). Among the children without bronchiolitis hospitalization during infancy, 14% developed asthma. In contrast, among those with hospitalization, 24% subsequently developed asthma. The Kaplan–Meier curves demonstrated a significant between-group difference in the rate of asthma (P_log-rank_ < 0.001; Figure 1). In the multivariable Cox proportional hazards model adjusting for 10 patient-level factors and patient clustering, compared with the no hospitalization group, the hospitalized group had a significantly higher rate of developing asthma (HR = 2.77, 95%CI = 2.24–3.43, *p* < 0.001, E-value = 3.43; Figure 2). In the complete case analysis, the findings did not materially change. For example, the hospitalized group had a significantly higher rate of asthma (HR = 2.11, 95%CI = 1.43–3.13, *p* < 0.001, E-value = 2.73; Figure S3).

The Kaplan–Meier curves in the overall analytic cohort (*n* = 11,762) showed that compared with the no bronchiolitis hospitalization group, the rate of developing asthma by the age of 12 years was significantly higher in the bronchiolitis hospitalization group (P_log-rank_ < 0.001). The corresponding hazard ratio estimates are presented in Figure 2.

### 3.3. Examination of Potential Effect Modification

**Figure 3:**
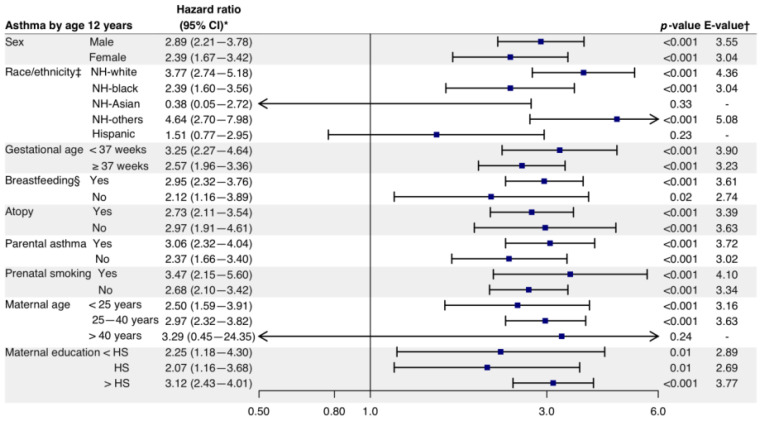
Effect of severe bronchiolitis on the development of asthma by major demographic and clinical factors. Arrows indicate that the 95% CI of the hazard ratio exceeds the lower or higher limit of the x-axis. * The Cox proportional hazard model was adjusted for 10 potential confounders (sex, race and ethnicity, calendar year of childbirth, gestational age, breastfeeding, child’s atopy, parental asthma, prenatal smoking, maternal age, and maternal education, except for the stratifying covariate), accounting for patient clustering by cohort. ^†^ The E-value (with its lower 95% CI bound) represents how strongly unmeasured confounder(s) would have to be associated with the exposure and outcome in order for the observed association to be independent. ^‡^ Test for the interactions between bronchiolitis hospitalization and demographic factors on the development of asthma: P_interaction_ = 0.02 for race and ethnicity. ^§^ Test for the interactions between bronchiolitis hospitalization and demographic factors on the development of asthma: P_interaction_ = 0.07 for breastfeeding. The *p*-values for the interactions of the other demographic factors were >0.10 and are listed in Table S3. Abbreviations: CI, confidence interval; HS, high school; NH, non-Hispanic.

For the major demographic factors (Table S3), there was a significant interaction between bronchiolitis hospitalization and race and ethnicity on the rate of developing asthma (P_interaction_ = 0.02; Figure 3 and Figure S4B). The magnitude of the bronchiolitis–asthma association was greater in non-Hispanic White (HR = 3.77, 95%CI = 2.74–5.18, *p* < 0.001, E-value = 4.36) and non-Hispanic Black (HR = 2.39, 95%CI = 1.60–3.56; *p* < 0.001, E-value = 3.04) children compared with Hispanic children (HR = 1.51, 95%CI = 0.77–2.95, *p* = 0.23). There was no significant interaction for non-Hispanic Asian or non-Hispanic other races.

With regard to clinical factors, there was a non-significant interactive effect between bronchiolitis hospitalization and breastfeeding on the rate of developing asthma (P_interaction_ = 0.07). The magnitude of the association was greater in the breastfeeding group (HR = 2.95, 95%CI = 2.32–3.76, *p* < 0.001, E-value = 3.61) compared with the non-breastfeeding group (HR = 2.12, 95%CI = 1.16–3.89, *p* = 0.02; Figure 3 and Figure S4D). While there was no significant effect modification by other clinical factors, the severe bronchiolitis–asthma association remained significant in most strata (e.g., children with parental asthma, HR = 3.06, 95%CI = 2.32–4.04, *p* < 0.001, E-value = 3.72 vs. children without parental asthma, HR = 2.68, 95%CI = 2.10–3.42, *p* < 0.001, E-value = 3.02 (Figure 3). Furthermore, in most strata, the magnitude of the association was stronger in bronchiolitis hospitalization than in the other demographic and clinical factors (Figure S4A–H).

## 4. Discussion

In this analysis of nationwide pediatric cohort data of 11,762 infants, we found that infants hospitalized for bronchiolitis had a significantly higher rate of developing asthma by the age of 12 years compared with those without. Additionally, we also observed that the magnitude of the association was stronger for bronchiolitis hospitalization than for the other demographic and clinical factors in most strata. Furthermore, there was a quantitative effect modification by race and ethnicity and possibly breastfeeding on the rate of asthma development. To the best of our knowledge, this is the first nationwide investigation that has demonstrated the longitudinal relationship of severe bronchiolitis during infancy with incident asthma and its heterogeneity by subpopulations.

Acute respiratory infection in early childhood (e.g., infant bronchiolitis) has been considered a major risk factor for incident asthma for decades [35,36,37,38,39]. In agreement with our findings, previous epidemiological data have found that 20–40% of infants hospitalized for bronchiolitis subsequently develop asthma in childhood [12,17,26,27,28]. Recently, several studies have suggested that the asthma risk may be lower than was suggested in early studies, but the risk still remains significantly higher than expected in the general pediatric population. For example, a birth cohort study in Boston reported that 27% of infants with severe bronchiolitis later developed asthma, compared with 12% in the general population [11]. Additionally, a multicenter study has shown that children hospitalized for RSV bronchiolitis in the first 2 years of life had a 22% prevalence of asthma at 6 years [24]. The apparent discrepancies between these reports may be attributable to the differences in the study design, setting, sample, outcome definition, or any combination of these factors. The current large-scale study built on these earlier reports and extended them by demonstrating the longitudinal relationship of severe bronchiolitis with the asthma risk in a demographically and geographically diverse nationwide sample.

The literature has attributed the mechanisms underlying the associations between bronchiolitis and the development of asthma to severe virus (e.g., RSV, rhinovirus) infection [17,40,41,42], host genetics [24,43,44,45], Type 2 airway inflammation [40,46,47,48,49], the airway microbiome [50,51,52], and a complex interplay of these factors [53,54,55,56,57] in infancy, which is a critical period of lung and immune development. However, the observed heterogeneity by clinical factors (e.g., race and ethnicity) warrants further investigation. First, previous studies demonstrated that the incidence and prevalence of asthma are high in non-Hispanic Black and Puerto Rican children [1,2,3,4,58]. In contrast, the current analysis found that the magnitude of the bronchiolitis–asthma association was significantly greater in non-Hispanic White and non-Hispanic Black children than in Hispanic children. This discrepancy suggests that risk factors other than severe bronchiolitis (e.g., genetic factors, socioeconomic status, and/or environmental factors [5,18,59]) may have contributed to the observed effect modification by race and ethnicity. Second, the current study also found non-significant (P_interaction_ = 0.07) heterogeneity in the bronchiolitis–asthma link by breastfeeding status. To date, the literature on the role of breastfeeding in the development of asthma has been conflicting, with breastfeeding having protective [19,60,61,62] or null [63,64] effects. Breast milk provides early passive immunity through the biological activity of immunoglobulins such as IgG, IgM, and secretory IgA [65,66,67,68]. However, breast milk also contains factors that actively regulate the infant’s immune system (e.g., Type 2 cytokines (IL-4, IL-5, and IL-13) and chemokines) [19,65,67,69]. Little remains known about the exact interplay among early-life virus infection, breastfeeding, infant immune function, and the subsequent development of childhood asthma. Notwithstanding this complexity, the heterogeneity of the bronchiolitis–asthma relationship across different demographical and clinical subpopulations is important. These results will facilitate further investigations to identify the subgroups at highest risk and their underlying mechanisms, and thereby advance the development of targeted prevention strategies for childhood asthma.

## 5. Limitations

Our study has several potential limitations. First, some of the longitudinal cohorts were not eligible for the current analysis because of the exclusion criteria, e.g., a lack of exposure and outcome data. Second, our study did not include detailed viral testing data during hospitalization for bronchiolitis. However, bronchiolitis is a clinical entity, and RSV (50–80%) and rhinovirus (20–30%) are two major viral causes of bronchiolitis in the first year of life [70,71]. While we did not have data on the viral etiology of bronchiolitis, infant bronchiolitis is a clinical diagnosis and was considered as such in our analyses. Third, a detailed classification of Hispanic ethnicity (e.g., Puerto Rican versus Mexican) was not ascertained in the present study, which is potentially important, since Puerto Rican children have a much higher rate of asthma compared with Mexican and other Hispanic children [1,4,58]. Fourth, severe bronchiolitis during infancy could contribute to the development of specific phenotype(s) of childhood asthma, but asthma phenotypes were not evaluated in this study. Fifth, as with any observational study, our causal inference may have been confounded by unmeasured factors, such as host genetics. Regardless, the estimated E-values support the robustness of the reported inferences. Lastly, some of the cohort children are at a high risk for asthma (e.g., those with a parental history of asthma and/or allergy). Therefore, our inferences should be carefully generalized to the general pediatric population.

## 6. Conclusions

Based on data from nationwide pediatric cohorts of 11,762 infants, we found that bronchiolitis hospitalization during infancy is associated with a significantly higher risk of developing childhood asthma. Our data extend prior research by offering greater generalizability and demonstrating quantitative heterogeneity by individual characteristics, such as race and ethnicity and possibly breastfeeding status. We also note that the severe bronchiolitis–asthma association was present in many population subgroups. For researchers, our data should facilitate further investigations into the mechanisms underlying the links among infant bronchiolitis, demographic and clinical factors, and the development of asthma. For clinicians, our findings not only provide an evidence base for early identification of the children at high risk for asthma but also offer opportunities for early preventive interventions in this large high-morbidity population.

## Figures and Tables

**Table 1:** Baseline characteristics of the cohort infants, by bronchiolitis hospitalization (severe bronchiolitis).

	Overall (*n* = 11,762)	No Bronchiolitis Hospitalization Group (*n* = 10,632; 90%)	Bronchiolitis Hospitalization Group (*n* = 1130; 10%)
Child characteristics			
Age (month), median (IQR)	9 (7−11)	9 (7−12)	5 (5−5)
Male sex	6214 (53%)	5536 (52%)	678 (60%)
Race and ethnicity			
Non-Hispanic white	6219 (53%)	5713 (54%)	506 (45%)
Non-Hispanic black	2141 (18%)	1925 (18%)	216 (19%)
Non-Hispanic Asian	220 (2%)	200 (2%)	20 (2%)
Non-Hispanic other	996 (9%)	917 (9%)	79 (7%)
Hispanic	2111 (18%)	1805 (17%)	306 (27%)
Gestational age ≥ 34 weeks	8888 (87%)	7905 (86%)	983 (93%)
Birth weight (g), mean (SD)	3056 (922)	3048 (939)	3127 (737)
Low birth weight infant (<2500 g)	1869 (17%)	1708 (17%)	161(15%)
Perinatal breastfeeding	5638 (85%)	4921 (87%)	717 (78%)
Atopy *	2632 (47%)	2499 (53%)	133 (14%)
Parental history of asthma	3404 (36%)	3024 (36%)	380 (35%)
Maternal characteristics			
Prenatal smoking	1247(13%)	1101 (13%)	146 (14%)
Maternal age at delivery (years)			
<25	2491 (23%)	2443 (23%)	48 (18%)
25−40	7943 (74%)	7737 (74%)	206 (79%)
>40	240 (2%)	234 (2%)	6 (2%)
Maternal education			
<High school	769 (7%)	748 (7%)	21 (8%)
High school	1433 (14%)	1397 (14%)	36 (14%)
College or above	8335 (79%)	8132 (79%)	203 (78%)

Abbreviations: IQR, interquartile range; SD, standard deviation * Including healthcare provider-diagnosed eczema, food allergy, and allergic rhinitis.

## Data Availability

Not applicable.

## Supplementary Materials

The following supporting information can be downloaded at https://www.mdpi.com/article/10.3390/biomedicines11010023/s1. Table S1: Principal investigators (PIs) of the 53 participating cohorts in the ECHO program; Table S2: The number of missing data for each of major variables; Table S3: Test for interactions between bronchiolitis hospitalization during infancy and major demographic factors on the risk of asthma development; Figure S1: Locations of enrollment cohorts for children included in the analyses; Figure S2: Study flow; Figure S3: Associations of bronchiolitis hospitalization in infancy with the subsequent development of asthma (complete case analysis); Figure S4: Stratified analysis for the multivariable associations of bronchiolitis hospitalization during infancy with the development of asthma by major demographic and clinical factors.

## Abbreviations

CI	confidence interval
DAC	Data Analysis Center
ECHO	Environmental influences on Child Health Outcomes
HR	hazard ratio
IgA	immunoglobulin A
IgG	immunoglobulin G
IgM	immunoglobulin M
IL	interleukin
IQR	interquartile range
IRB	institutional review board
NIH	National Institutes of Health
RSV	respiratory syncytial virus
