# Supplementary material for: Association of Severe Bronchiolitis during Infancy with Childhood Asthma Development: An Analysis of the ECHO Consortium
Source: Biomedicines. 2022 Dec 22;11(1):23. doi: 10.3390/biomedicines11010023 (PMC9855570; doi:10.3390/biomedicines11010023)
Supplement: Supplementary file 1 [file biomedicines-11-00023-s001.zip › biomedicines-2080589-supplementary.pdf]

SUPPLEMENTARY ONLINE CONTENT

Association of severe bronchiolitis during infancy with childhood asthma development: An analysis of the ECHO consortium

Authors: Makiko Nanishi MD; Aruna Chandran MD, MPH; Xiuhong Li MAS; Joseph B. Stanford MD, MSPH; Akram N. Alshawabkeh PhD; Judy L. Aschner MD, PhD; Dana Dabelea MD, PhD; Anne L. Dunlop MD, MPH; Amy J. Elliott PhD; James Gern MD; Tina Hartert, MD, MPH; Julie Herbstman PhD; Gurjit K. Khurana Hershey, MD, PhD; Alison E. Hipwell PhD, ClinPsyD; Margaret R. Karagas PhD; Catherine J. Karr MD, PhD; Leslie D. Leve PhD; Augusto A. Litonjua, MD, MPH ;Cindy T. McEvoy MD; Rachel L. Miller MD; Emily Oken MD, MPH; T. Michael O'Shea MD, MPH; Nigel Paneth MD, MPH; Scott T. Weiss MD; Robert O. Wright, MD MPH; Rosalind J. Wright MD, MPH; Kecia N. Carroll MD MPH; Xueying Zhang PhD; Qi Zhao; Edward Zoratti MD; Carlos A. Camargo Jr. MD, DrPH; Kohei Hasegawa MD, MPH, PhD and Environmental Influences on Child Health Outcomes (ECHO) collaborators

Contents

Table S1. Principal investigators (PIs) of the 53 participating cohorts in the ECHO program
Table S2. The number of missing data for each of major variables
Table S3. Test for interactions between bronchiolitis hospitalization during infancy and major demographic factors on the risk of asthma development
Figure S1. Locations of enrollment cohorts for children included in the analyses
Figure S2. Study flow
Figure S3. Associations of bronchiolitis hospitalization in infancy with the subsequent development of asthma (complete case analysis)
Figure S4. Stratified analysis for the multivariable associations of bronchiolitis hospitalization during infancy with the development of asthma by major demographic and clinical factors

Table S1. Principal investigators (PIs) of the 53 participating cohorts in the ECHO program

Cohort PI	Cohort name
Akram Alshawabkeh, PhD	ECHO in Puerto Rico
Gloria Pryhyber, MD	Prematurity and Respiratory Outcomes Program (DINE-PROP)
Roberta Keller, MD	Trial of Late Surfactant (TOLSURF)
Annemarie Stroustrup, MD, MPH	NICU Hospital Exposures and Long-Term Health (DINE-NICUHEALTH)
Dennis Mayock, MD	Preterm Erythropoietin Neuroprotection Trial (DINE-PENUT)
Clancy Blair, MPH, PhD	Family Life Project (FLP)
Carlos Camargo, MD, DrPH	The 35th Multicenter Airway Research Collaboration (MARC-35) cohort
Carlos Camargo, MD, DrPH	The 43rd Multicenter Airway Research Collaboration (MARC-43) cohort
Dana Dabelea, MD, PhD	The Healthy Start Study
Sean Deoni, PhD	Myelination and Behavior across Maturation (BAMBAM) study
Cristiane Duarte, MPH, PhD	The Boricua Youth Study (BYS)
Anne Dunlop, MD, MPH	Atlanta ECHO Cohort of Emory University
Amy Elliott, PhD	The Safe Passage Study
Assiamira Ferrara, MD, PhD	Pregnancy Environment and Lifestyle Study (PETALS)
Lisa Croen, PhD	Kaiser Permanente Research Bank (KPRB)
Edward Zoratti, MD	Microbes, Allergy, Asthma & Pets Study (MAAP)
Christine Seroogy, MD	Wisconsin Infant Study Cohort (WISC)
Leonard Bacharier, MD	Urban Environment and Childhood Asthma (URECA)
Neeru Hershey, MD, PhD	Cincinnati Childhood Allergy & Air Pollution Study (CCAAPS)
Tina Hartert, MD, MPH	Infant Susceptibility to Pulmonary Infections and Asthma Following RSV Exposure (INSPIRE)
Christine Johnson, MPH, PhD	Wayne County Health Environment Allergy and Asthma (WHEALS)
Irva Picciotto, MPH, PhD	Revising the CHARGE Study children (ReCHARGE)
Alison Hipwell, PhD	Pittsburgh Girls Study
Margaret Karagas, PhD	New Hampshire Birth Cohort Study
Frances Tylavsky, DrPH	Conditions Affecting Neurocognitive Development and Learning in Early Childhood (CANDLE) Study
Sheela Sathyanarayana, MD, MPH	Global Alliance to Prevent Prematurity and Stillbirth (GAPPS) study
Barry Lester, PhD	Neonatal Neurobehavior and Outcomes in Very Preterm Infant (ECHO-NOVI)
Leslie Leve, PhD	Early Growth and Development Study (EGDS-CI)
Jenae Neiderhiser, PhD	Early Growth and Development Study - Cohort II

Leslie Leve, PhD	Early Parenting of Children
Scott Weiss, MD	The Vitamin D Antenatal Asthma Reduction Trial (VDAART)
Cynthia McEvoy, MD	Vitamin C to Decrease Effects of Smoking in Pregnancy on Infant Lung Function (VCSIP)
Cynthia McEvoy, MD	In-Utero Smoke, Vitamin C, and Newborn Lung Function
Rebecca Landa, PhD	Kennedy Krieger Institute, the Baby Siblings Research Consortium (BSRC)
Sally Ozonoff, PhD	University of California Davis - BSRC
Rebeccca Schmidt, PhD	University of California, Markers of Autism Risk in Babies- Learning Early Signs (MARBLES)
Joseph Piven, MD	Infant Brain Imaging Study (IBIS)
Lisa Croen, PhD	Early Autism Risk Longitudinal Investigation (EARLI)
Emily Oken, MD, MPH	The Project Viva cohort
Mike O'Shea, MD, MPH	Extremely Low Gestational Age Newborn (ELGAN) study
Nigel Paneth, MD, MPH	Archive for Research in Child health (ARCH)
Julie Herbstman, PhD	Sibling Cohort
Joseph Stanford, MD, MSPH	National Childrens Study IVS Salt Lake County (NCS-IVS)
Joseph Stanford, MD, MSPH	Alternate Recuitment Strategu Cache County (NCS-ARS)
Christy Porucznik, PhD, MSPH	Home Observation of Periconceptional Exposures Utah (HOPE-Utah)
Robert Silver, MD	Nulliparous Pregnancy Outcomes Study: Monitoring Mothers to be (NuMoM2B-Utah)
Joseph Stanford, MD, MSPH	Time to Pregnancy - Utah (TTP-Utah)
Elisabeth Conradt, PhD	Baby Affect & behacior Study (BABY-Utah)
Rosalind Wright, MD, MPH	Asthma Coalition on Community, Environment & Social Stress (ACCESS)
Rosalind Wright, MD, MPH	PRogramming of Intergenerational Stress Mechanisms
Kathi Huddleston, PhD	First 1000 Days Inova Childhood Longitudinal Study
Catherine Karr, MD, PhD, Leonard Trasande, MD, MPP	The Infant Development and Environment Study (TIDES)
Frederica Perera, MPH, DrPH, PhD	Columbia Center for Children's Environmental Health (CCCEH) / Mothers and Newborns (M and N)

Table S2. The number of missing data for each of major variables

	**The number of missing data**
**Child characteristics**	
Sex	0
Race and ethnicity	75 (<1%)
Gestational age	1516 (13%)
Perinatal breastfeeding	5158 (44%)
Atopy*	6155 (52%)
Parental history of asthma	2260 (19%)
**Maternal characteristics**	
Prenatal smoking	2151 (18%)
Maternal age at delivery	1088 (9%)
Maternal education	1225 (10%)

* Including healthcare provider-diagnosed eczema, food allergy, and allergic rhinitis

Table S3. Test for interactions between bronchiolitis hospitalization during infancy and major demographic factors on the risk of asthma development

**Demographic factor**	**$P_{interaction}$**
Sex	0.91
Race and ethnicity	0.02
Gestational age	0.54
Breastfeeding	0.07
Atopy*	0.34
Parent asthma	0.89
Parent smoking	0.45
Maternal age at delivery	0.60

* Including healthcare provider-diagnosed eczema, food allergy, and allergic rhinitis

Figure S1. Locations of ECHO enrollment cohorts included in the current analyses

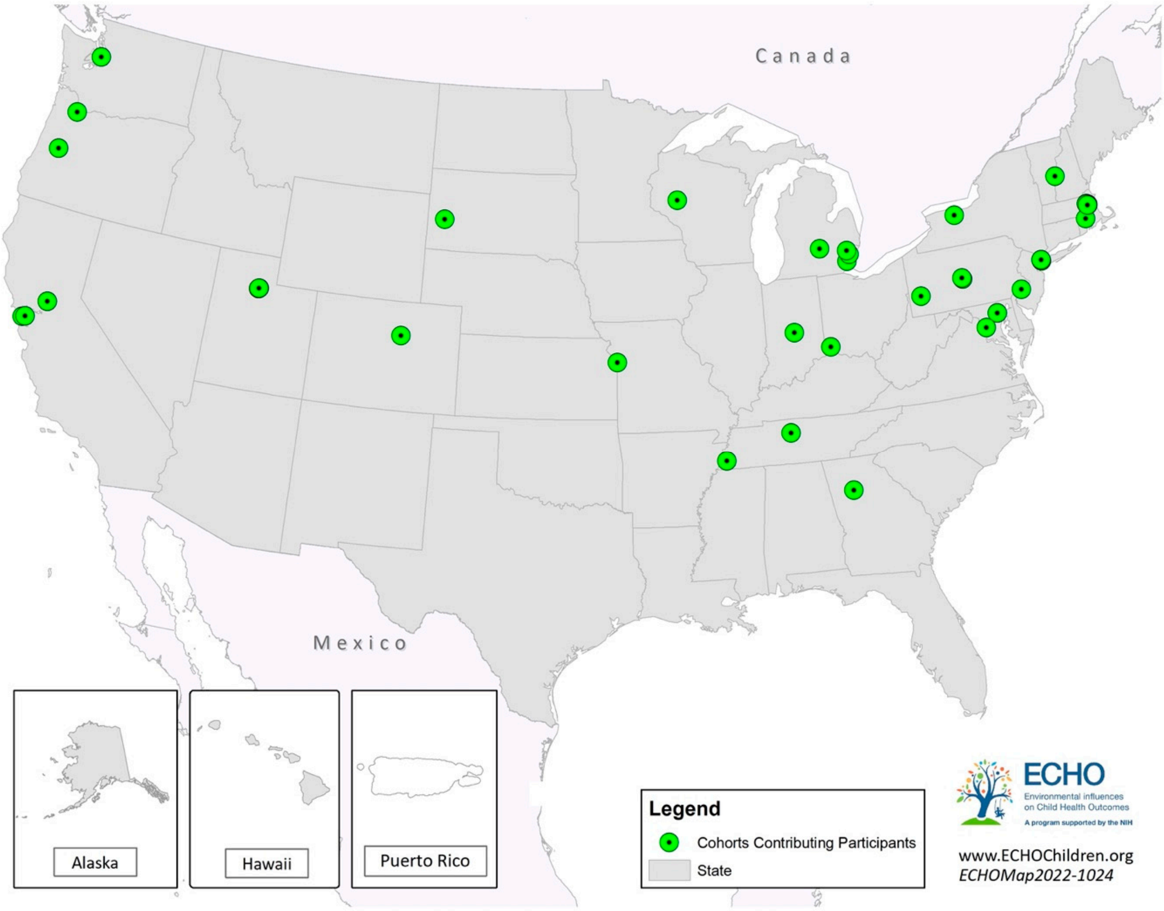

Figure S2. Study flow

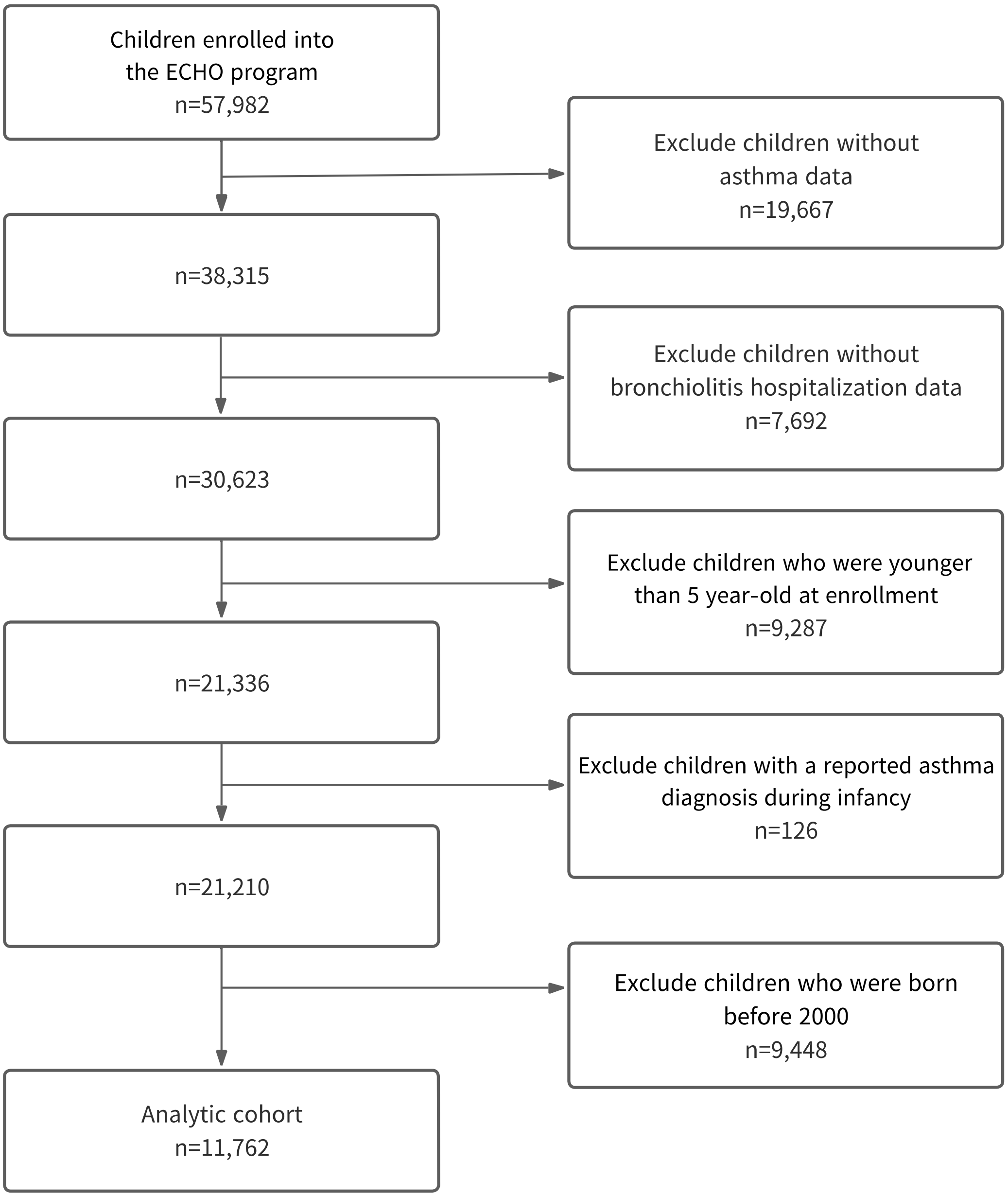

Figure S3. Associations of bronchiolitis hospitalization in infancy with the subsequent development of asthma (complete case analysis)

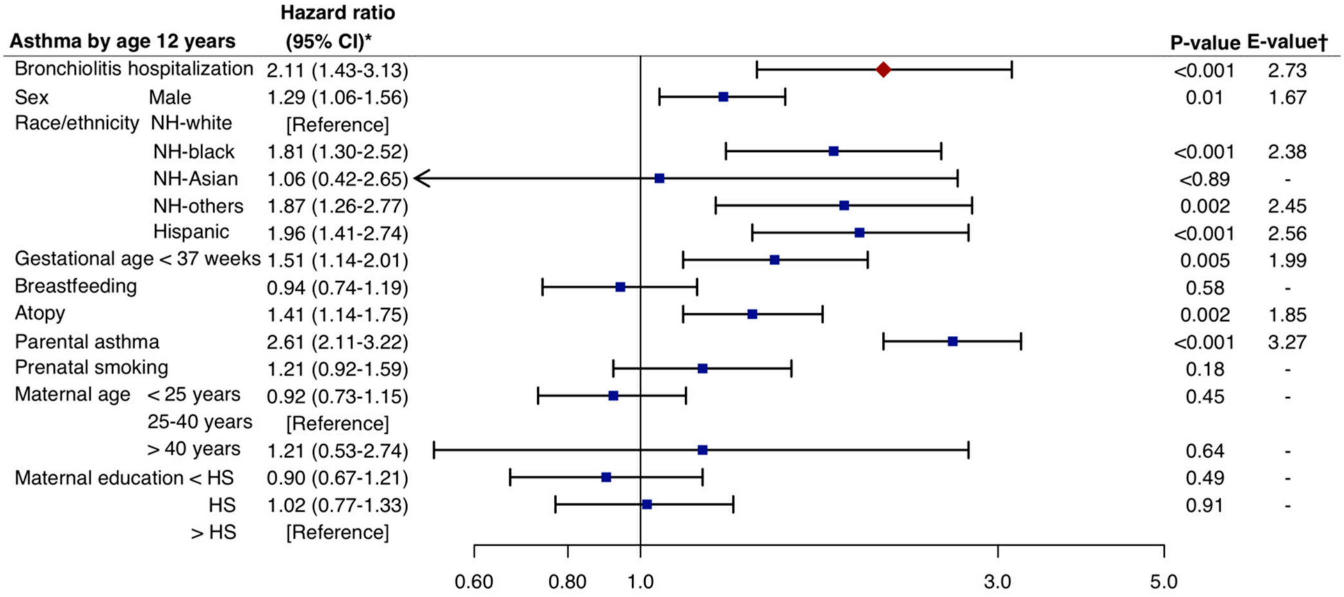

Figure S4. Stratified analysis for the multivariable association of bronchiolitis hospitalization during infancy with the development of asthma by major demographic and clinical factors

A) Stratification by sex

A-1) Male

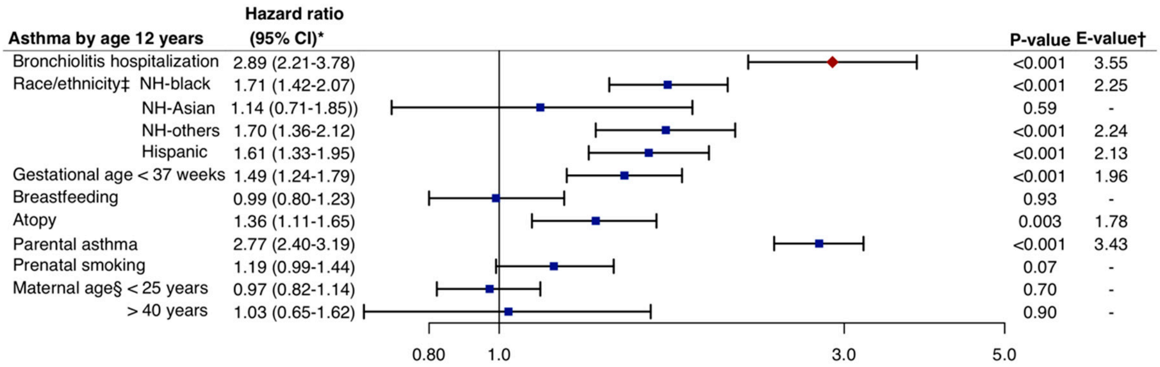

A-2) Female

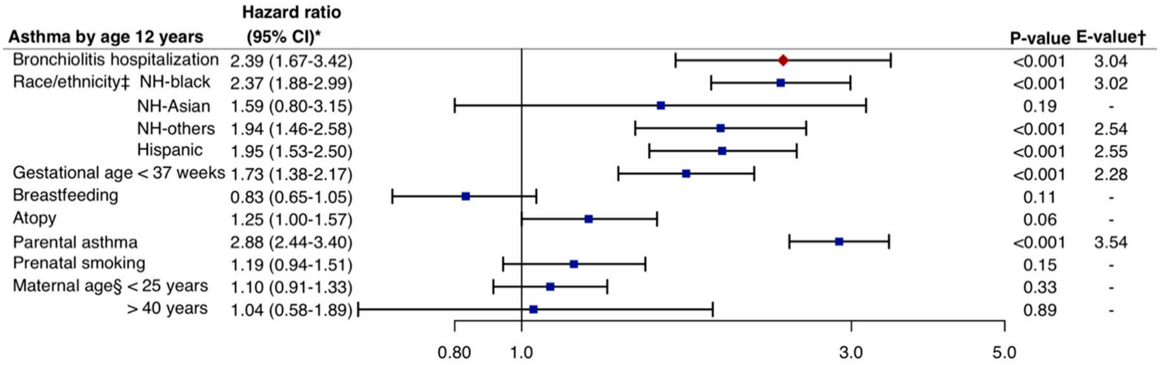

B) Stratification by race and ethnicity

B-1) Non-Hispanic white

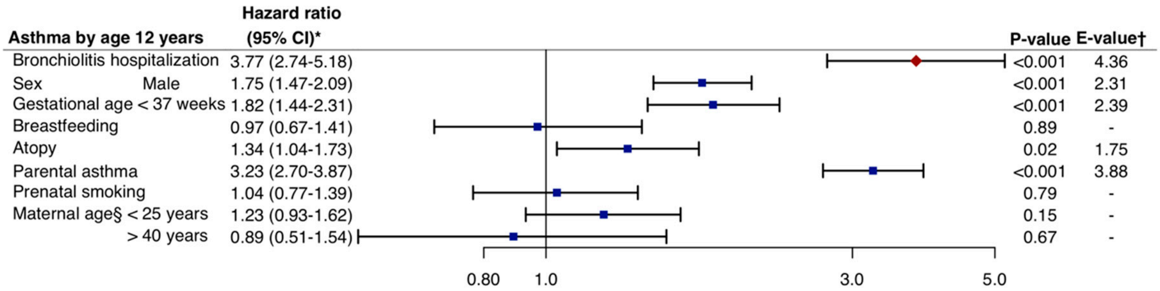

B-2) Non-Hispanic black

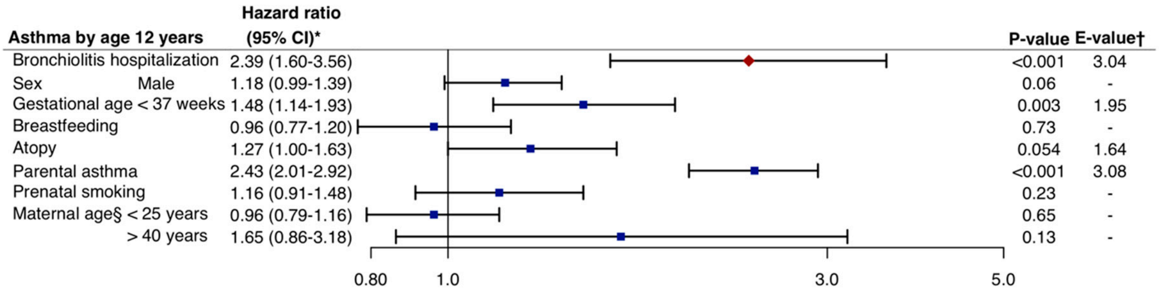

B-3) Non-Hispanic Asian

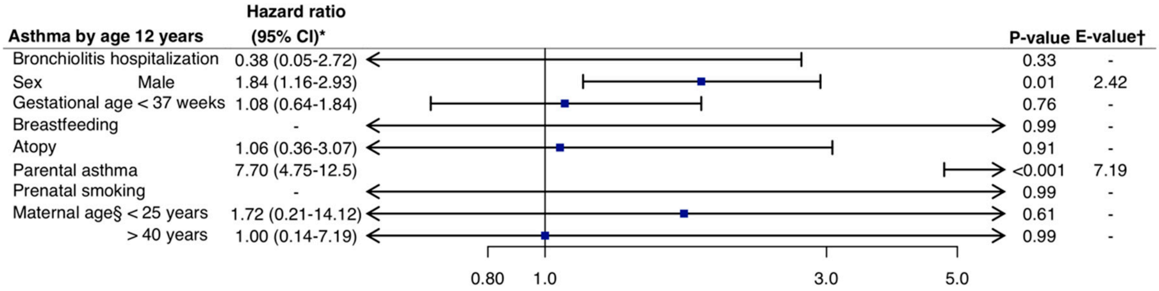

B-4) Non-Hispanic others

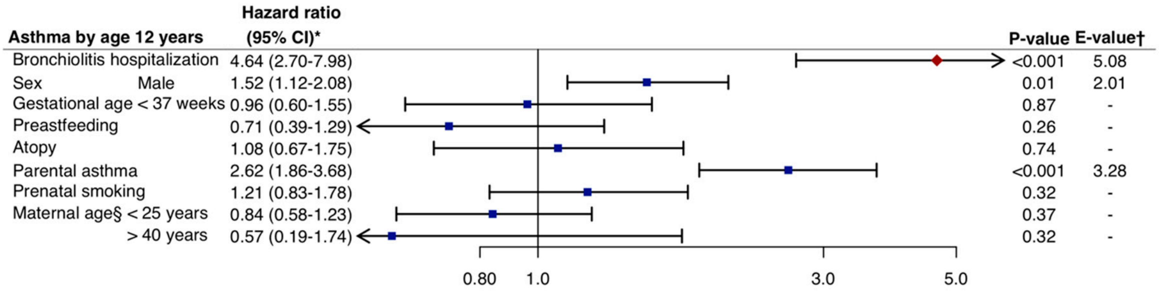

B-5) Hispanic

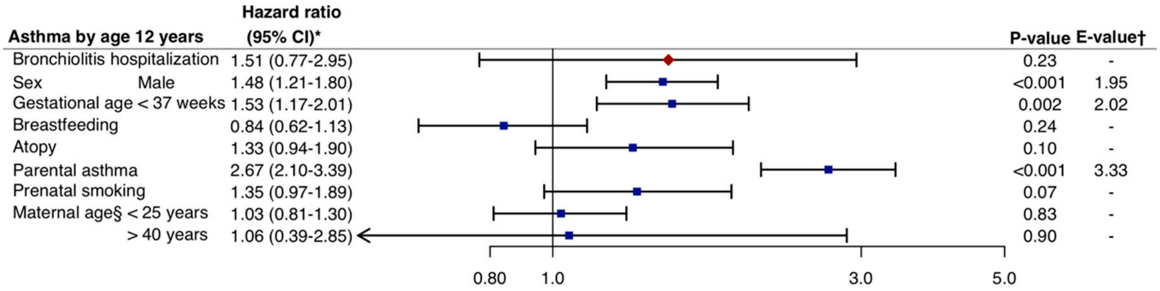

C) Stratification by gestational age

C-1) Gestational age < 37 weeks

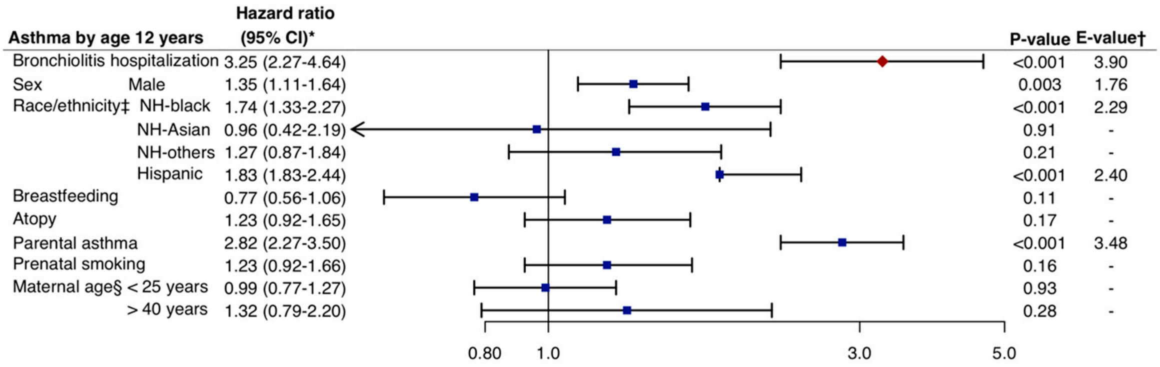

C-2) Gestational age ≥ 37weeks

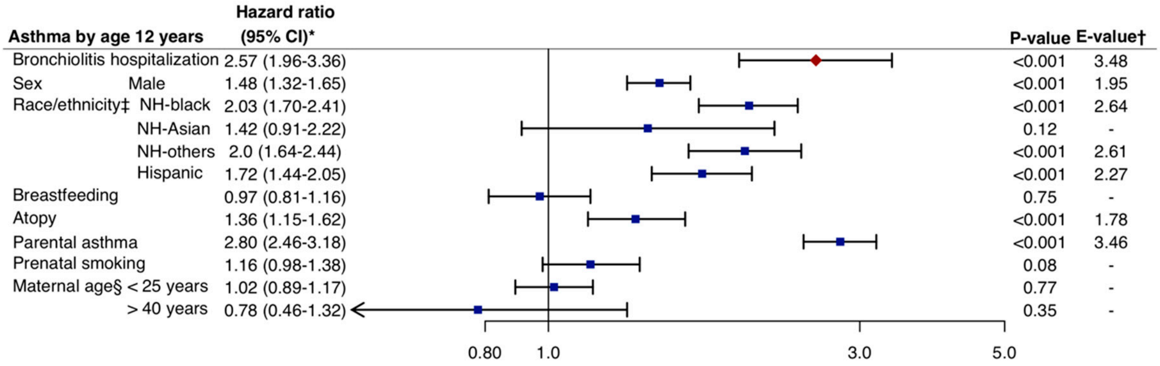

D) Stratification by breastfeeding

D-1) With breastfeeding

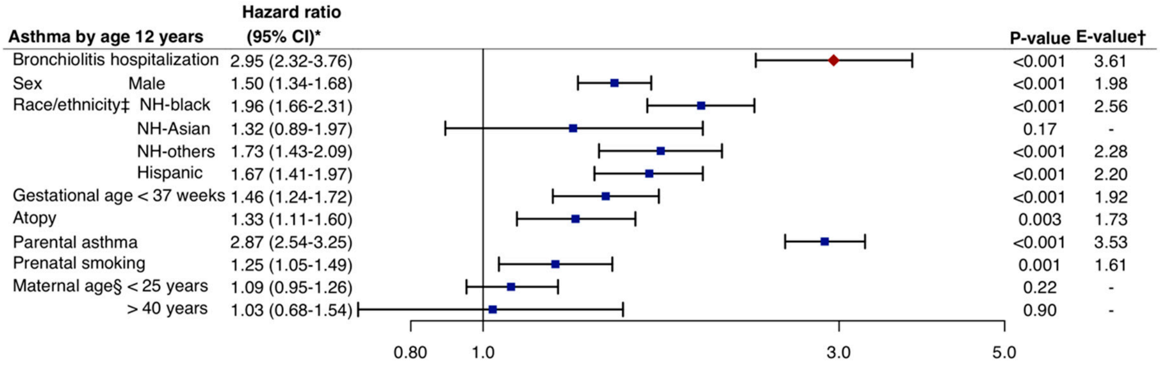

D-2) Without breastfeeding

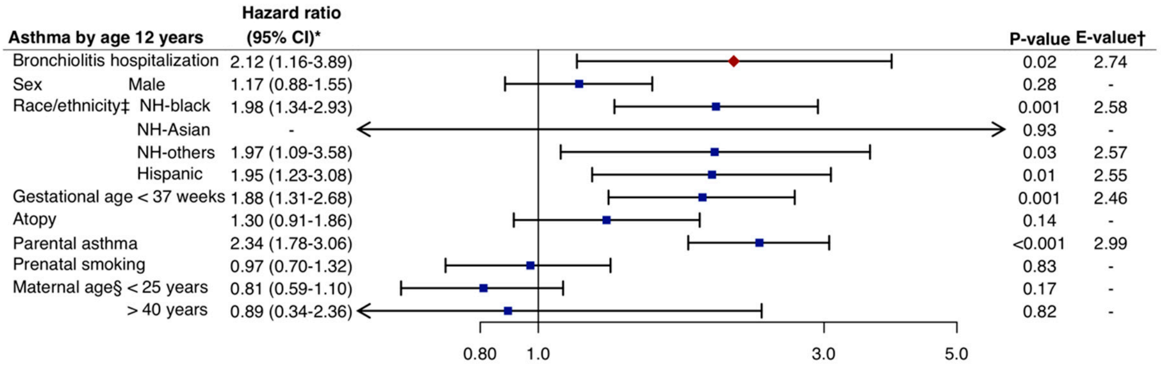

E) Stratification by atopy

E-1) With atopy

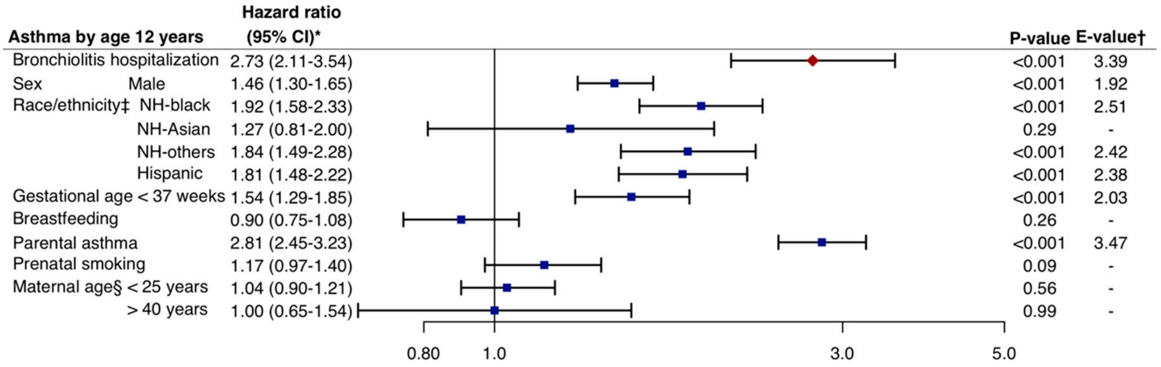

E-2) Without atopy

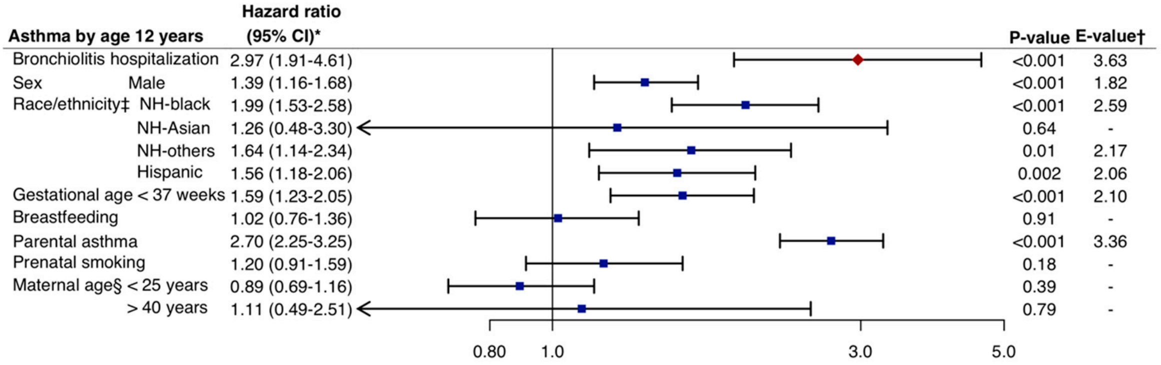

F) Stratification by parental asthma

F-1) With parental asthma

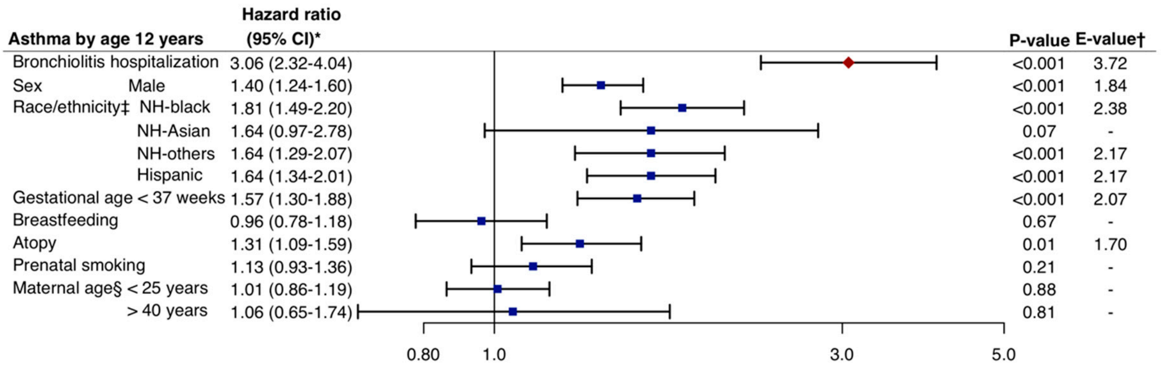

F-2) Without parental asthma

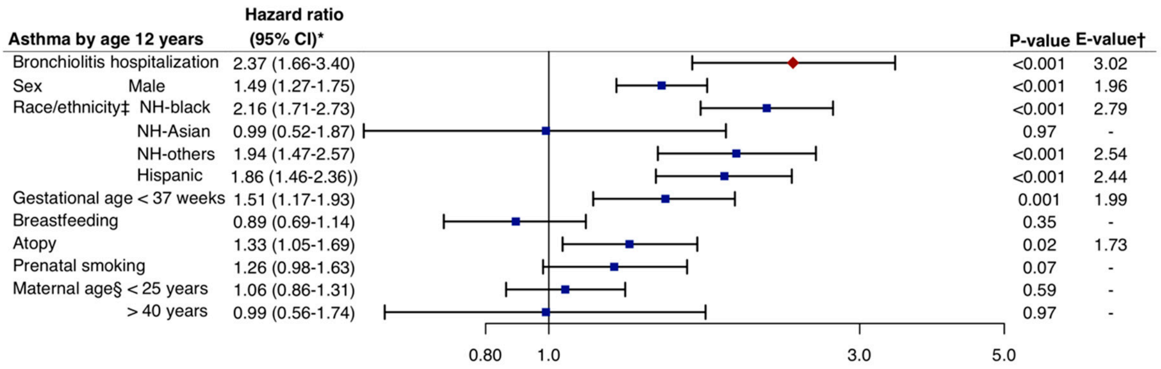

G) Stratification by prenatal smoking

G-1) With prenatal smoking

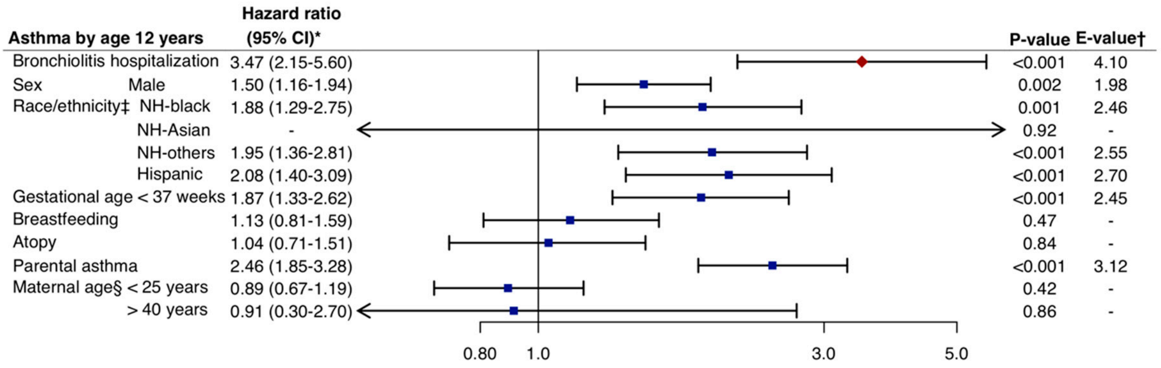

G-2) Without prenatal smoking

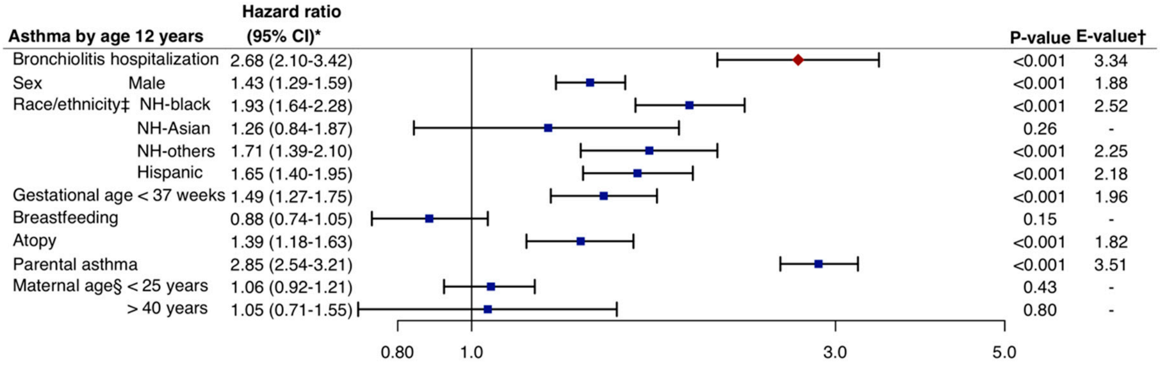

H) Stratification by maternal age

H-1) Maternal age < 25 years

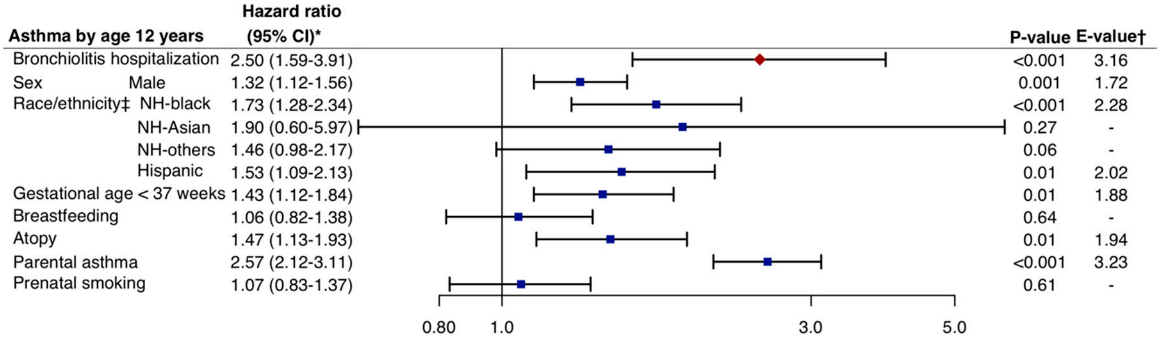

H-2) Maternal age 25-40 years

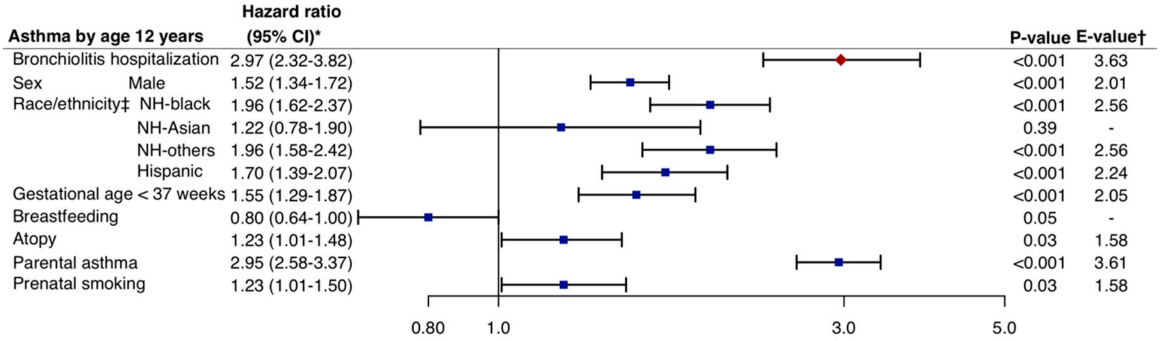

H-3) Maternal age > 40 years

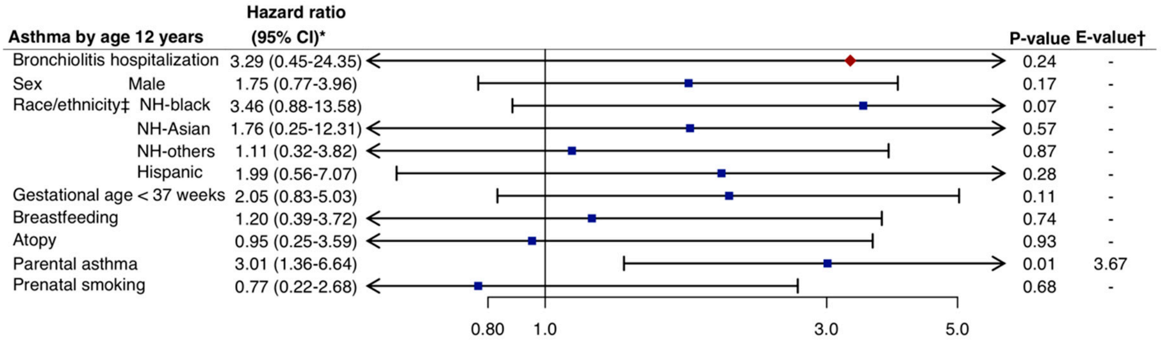

Arrows indicate that the 95% CI of the hazard ratio exceeds the lower or higher limit of the x-axis. The results of are not reported in these figures.

* Cox proportional hazards models adjusted for the listed covariates, maternal education, and calendar year, with accounting for patient clustering by cohort

† The E-value (with its lower 95% CI bound) represents how strongly unmeasured confounder(s) would have to be associated with the exposure and outcome in order for the observed association to be independent.

‡ The reference group is non-Hispanic white.

§ The reference group is 25-40 years.

Abbreviations: CI, confidence interval; NH, Non-Hispanic
